# Mutational change of CTX‐M‐15 to CTX‐M‐127 resulting in mecillinam resistant *Escherichia coli* during pivmecillinam treatment of a patient

**DOI:** 10.1002/mbo3.941

**Published:** 2019-10-01

**Authors:** Karen Leth Nielsen, Katrine Hartung Hansen, Jesper Boye Nielsen, Jenny Dahl Knudsen, Kristian Schønning, Niels Frimodt‐Møller, Frederik Boëtius Hertz, Filip Jansåker

**Affiliations:** ^1^ Department of Clinical Microbiology Rigshospitalet Copenhagen Denmark; ^2^ Department of Clinical Microbiology Hvidovre Hospital Hvidovre Denmark; ^3^ Department of Clinical Medicine Faculty of Health and Medical Sciences University of Copenhagen Copenhagen Denmark; ^4^ Department of Clinical Microbiology Herlev Hospital Herlev Denmark; ^5^ Department of Virus and Microbiological Special Diagnostics Statens Serum Institut Denmark

**Keywords:** *Escherichia coli*, LPS, mecillinam, metabolism, mutation, O‐antigen, Pivmecillinam, resistance, serotype, whole‐genome sequencing

## Abstract

Pivmecillinam (amdinocillin pivoxil) is the recommended first‐choice antibiotic used to treat urinary tract infections (UTIs) in Denmark. The frequency of mutation to mecillinam (MEC) resistance is described as high in vitro; however, treatment of UTI has a good clinical response and prevalence of mecillinam resistance in *Escherichia coli* remains low despite many years of use. We describe occurrence of in vivo mecillinam resistance in a clinical isolate of ESBL‐producing *E. coli* following pivmecillinam treatment. The identified phenotypic differences in the mecillinam resistant isolate compared with the original mecillinam susceptible isolate were a full‐length LPS with O‐antigen (O25), mecillinam resistance and a lower MIC for ceftazidime. Regarding genotype, the resistant isolate differed with a mutation in *bla*_CTX‐M‐15_ to *bla*_CTX‐M‐127_, loss of a part of a plasmid and a genomic island, respectively, and insertion of a transposase in *wbbL*, causing the rough phenotype. The observed mecillinam resistance is expected to be caused by the mutation in *bla*_CTX‐M‐15_ with additional contribute from the serotype shift. We continue to recommend the use of pivmecillinam as first‐line treatment for UTI.

## Graphical abstract

In this study, we investigated the genetics behind mecillinam resistance which occurred in an extended‐spectrum β‐lactamase‐producing *Escherichia coli* during pivmecillinam treatment of a patient. The genomic comparison of the two isolates points toward a mutational change of blaCTX‐M‐15 into blaCTX‐M‐127 along with a serotype shift from rough to full‐length O25 as the mechanisms contributing to the change in mecillinam susceptibility.

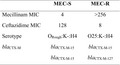

## 1 INTRODUCTION

Pivmecillinam (amdinocillin pivoxil) is the recommended first‐choice antibiotic used to treat urinary tract infections (UTIs) in Denmark. Yet, in laboratory settings, the frequency of mutation to mecillinam (MEC) resistance has been found to be very high (Thulin, Sundqvist, & Andersson, 2015). The clinical relevance of in vivo resistance is unknown, and previous studies of mecillinam for treatment of UTI found good clinical response (Titelman, Iversen, Kalin, & Giske, 2012). This could imply that most *Escherichia coli* with in vivo development of mecillinam resistance cannot survive in the bladder, as previously shown in vitro (Thulin et al., 2015). A study from France found a mutation in the promotor of a plasmid‐borne *bla*_TEM‐1_ gene to cause in vivo mecillinam resistance, yet the clinical relevance was unknown and combining mecillinam with amoxicillin/clavulanate inhibited the newly developed resistance (Birgy et al., 2017). In Denmark, we have seen a low prevalence of mecillinam resistance in *E. coli* with 7% among urine isolates in Danish hospitals and 6% of primary care urine isolates, despite many years of use (DANMAP, 2016). The resistance mechanisms reported from in vitro studies include a large variety of mutations in genes associated with, for example energy metabolism and LPS synthesis (Thulin et al., 2015). Thus, mecillinam resistance is difficult to detect based on genomic data.

In this study, we describe the development of mecillinam resistance following pivmecillinam treatment of a patient with complicated urinary tract infection caused by an extended‐spectrum β‐lactamase (ESBL)‐producing *E. coli* isolate sampled from a previous study by Jansåker, Frimodt‐Møller, Sjögren, and Dahl Knudsen (2014). Two clinical isolates without and with mecillinam resistance from the same patient were isolated before and after pivmecillinam treatment, respectively (MEC‐S and MEC‐R, respectively). The isolates caused complicated lower UTI in the patient, and resistance to mecillinam was observed, after treatment with pivmecillinam 400 mg *t.i.d* during the primary infection. The patient had recurrent symptoms of UTI, and the second urine sample, two weeks after the initial sample, revealed the same ESBL‐producing *E. coli*, however, now resistant to mecillinam.

## 2 MATERIALS AND METHODS

A range of phenotypic and genotypic characterizations were performed to characterize the isolates. With respect to phenotype, the isolates were subject to MIC determination using E‐test®, disk diffusion (EUCAST method) and serotyping. Additionally, the isolates were tested for synergy between mecillinam (10 µg) and ampicillin (10 µg) and amoxicillin/clavulanate (30 µg), respectively, by disk diffusion in order to evaluate the activity of mecillinam + ampicillin and mecillinam + amoxicillin/clavulanate in MEC‐S and MEC‐R. Phenotypic serotyping was performed by agglutination tests in microtiter plates using commercially available antisera against *E. coli* antigens (O:K:H) (SSI Diagnostica). The genotypes of the two isolates were investigated by whole‐genome sequencing (Miseq) based on paired‐end and mate‐pair Illumina libraries. SNP differences between the two isolates were identified with Geneious R9 using MEC‐S as reference for the SNP call followed by verification by inspection of reference mapping with parameters previously described (Nielsen et al., 2016). Presence/absence of genes was determined with GenAPI (Gabrielaite & Marvig, 2019) followed by read mapping of MEC‐R against MEC‐S (>10× coverage with unique mapping). For further description of the isolates, we used the following genomic tools: PHAST (phage identification, http://phast.wishartlab.com/), PlasmidFinder v2.1, ResFinder v3.2, MLST v2.0, SerotypeFinder v2.0 (O:H‐antigen) (https://cge.cbs.dtu.dk/services/). Any discrepancies between the isolates were verified in Geneious by read mapping.

## 3 RESULTS AND DISCUSSION

The phenotypic analyses proved identical MIC values between the two isolates for all tested antimicrobials, apart from mecillinam (4 → >256 µg/ml) and ceftazidime (128 → 8 µg/ml) (Table 1). The synergy test revealed that a combination of mecillinam + amoxicillin/clavulanate abolished resistance in MEC‐R, similar to the results from Birgy et al. (2017).

**Table 1:** Pheno‐ and genotypic data of MEC‐S and MEC‐R

Phenotype	MEC‐S		MEC‐R	
	MIC (µg/mL)	Interpretation^b^	MIC (µg/mL)	Interpretation^b^
Ampicillin	>256	R	>256	R
Mecillinam	4	S	>256	R
Cefpodoxime	>256	R	>256	R
Ceftazidime	128	R	8	R
Meropenem	0.032	S	0.032	S
Ciprofloxacin	>32	R	>32	R
Gentamicin	0.5	S	0.5	S
Tetracycline	4	S	4	S
Sulfamethoxazole	64	S	64	S
Trimethoprim	>32	R	>32	R
Nitrofurantoin	16	S	16	S
Piperacillin‐tazobactam	23^a^	S	25^a^	S
Serotype	O_Rough_:K‐:H4		O25:K‐:H4	

Genotype	MEC‐S	MEC‐R
Phylogroup	B2	B2
MLST	ST131	ST131
SeroTypeFinder	O25:K‐:H4	O25:K‐:H4
Scaffolds	16	19
Av. cov.	130	150
Inc‐groups	IncFIA, IncFIB, IncFII, IncX4, IncN, Col156	IncFIA, IncFIB, IncFII, IncX4, IncN
Resistance genes	*bla*_CTX‐M‐15_	*bla*_CTX‐M‐15_
	*bla*_CTX‐M‐15_	*bla*_CTX‐M‐127_
	*bla*_TEM‐1B_	*bla*_TEM‐1B_
	*bla*_TEM‐1B_	*bla*_TEM‐1B_
	*bla*_LAP‐2_ c	*bla*_LAP‐2_ c
	*QnrS1*	*QnrS1*
	*dfrA14*	*dfrA14*

a Zone diameter reported.

b Interpretation based on EUCAST breakpoints except for tetracycline and sulfamethoxazole where ECOFFs have been applied.

c Not in Resfinder database, but identified by BLAST.

Genomic analyses revealed no differences between the two isolates with respect to MLST (ST131) and phylogroup (B2) (Table 1). MEC‐R had one single nonsynonymous SNP compared with MEC‐S. This SNP was positioned in *bla*_CTX‐M‐15_, changing the beta‐lactamase to CTX‐M‐127 (A403G, N135D). In MEC‐S, both copies of *bla*_CTX‐M‐15_ were fully assembled. Oppositely, in MEC‐R, one of the genes was assembled correctly and the other had a contig break at the position where *bla*_CTX‐M‐15_ differs from *bla*_CTX‐M‐127_ (403 bp), most likely due to the SNP. Read mapping and normalized coverage (compared with average coverage across the whole genome) showed two full‐length *bla*_CTX‐M_ in MEC‐R, that is a *bla*_CTX‐M‐15_ and a b*la*_CTX‐M‐127_. This mutation was identified as common in vitro by Rosenkilde et al. (2019) after mecillinam selective pressure on a *bla*_CTX‐M‐15_ positive isolate and was associated with resistance to mecillinam and increased susceptibility for ceftazidime. *bla*_CTX‐M‐127_ has previously been identified in Danish surveillance of ESBL producing *E. coli* (DANMAP, 2016). Further studies are required to describe whether presence of *bla*_CTX‐M‐127_ is correlated to mecillinam treatment. We did not find any other mutations in the genes associated with mecillinam resistance in *E. coli* as previously reported (Birgy et al., 2017; Thulin et al., 2015; Titelman et al., 2012) when comparing MEC‐R with MEC‐S.

Regarding serotype, SeroTypeFinder identified O25:K‐:H2 in both MEC‐S and MEC‐R. However, from the phenotypic serotype, it was evident that MEC‐S was a rough isolate that did not produce the O25 antigen, but genetically, it belonged to the O25:K‐:H4 group (Table 1). SeroTypeFinder does not identify rough phenotypes. *wbbL* has previously been correlated with rough phenotypes so we compared this gene and the O‐antigen cluster of the two isolates by alignment in Geneious. The results showed that MEC‐R had a complete *wbbL*, whereas MEC‐S had a transposase inserted in *wbbL* disrupting the gene. We consider this the genetic cause of the rough serotype. Mutations in LPS have previously been described to affect mecillinam susceptibility in combination with other mutations (Antón, 1995). It is possible that this contributed to the increased MIC of mecillinam in MEC‐R, but this should be investigated further.

We performed GenAPI followed by visual inspection of read mappings of the GenAPI identified areas of the genomes. These analyses revealed two regions with differences between the genomes: (a) MEC‐R lacked a part of a plasmid (33,674 bp) encoding several hypothetical proteins, transposases and other mobile elements, iron regulation proteins as well as plasmid replicon Col156 (Top BLAST hit GenBank ID CP029577) and (b) MEC‐R lacked 44,258 bp of a genomic island situated on the chromosome similar to GEI II of Nissle 1917 (GenBank: AJ586888.1). The deleted part of GEI II included genes encoding antigen 43, a toxin/antitoxin system, hypothetical proteins and several genes from the K‐antigen cluster. As both isolates were phenotypically K‐, we do not expect the loss of capsule genes to affect the antibiotic susceptibility of the isolates. Neither of the missing regions are expected to contribute to the observed phenotypic susceptibility changes. The PHAST results were not conclusive, due to incomplete assembly of some of the phage contents (data not shown). Finally, the assembled genome of MEC‐S lacks one copy of *bla*_TEM‐1B_ compared with MEC‐R, but reference mapping confirmed similar normalized coverage of *bla*_TEM‐1B_ between the two isolates, and therefore, we conclude that both isolates carried two copies of *bla*_TEM‐1B_.

The MIC for mecillinam of the parental MEC‐S strain was 4 mg/L, which was below the breakpoint of 8 mg/L, but higher than the ECOFF (1 mg/L). Whether this lower susceptibility to mecillinam increased the risk for development of resistance after exposure to mecillinam should be investigated, since it could imply for a change of the clinical breakpoint for mecillinam.

## 4 CONCLUSIONS

In summary, here we describe clinical occurrence of mecillinam resistance in an ESBL‐producing *E. coli* following pivmecillinam treatment. We expect that the mutation in *bla*_CTX‐M‐15_ in combination with the serotype change caused the increased MIC for mecillinam and the lower MIC for ceftazidime.

Due to the low risk of resistance development, we continue to recommend the use of pivmecillinam as first‐line treatment for UTI.

## CONFLICT OF INTEREST

None declared.

## AUTHOR CONTRIBUTION

KN, KH, JN, JK, NFM, FJ and FBH: conceptualized the study; KN, KH, JN, KS, FBH, and FJ: involved in data curation; KN, KH, and FJ: involved in formal analysis; NFM: involved in funding acquisition; KN, KH, JN, JK, KS, NFM, FBH, and FJ: involved in investigation; KN, KH, JN, JK, KS, FBH, and FJ: contributed in methodology; KN, JK, and NFM: administered the project; NFM: contributed with resources; JK, KS, NFM, and FBH: involved in supervision; KS and KN: validated the study; KN, KH, JN, JK, KS, NFM, and FBH: contributed in preparing the original draft; KN, KH, JN, JK, KS, NFM, FBH, and FJ: reviewed and edited the article.

## ETHICAL APPROVAL

The study has been approved by the Danish Data Protection Agency (I –suitnr. 01755 and id.nr. HVH‐2012‐022) for a previously published study (Jansåker et al., 2014). Included patients were asked to participate and gave written consent prior to inclusion.

## Data Availability

The assembled genomes are available from Figshare: https://doi.org/10.6084/m9.figshare.9804566.v1
